# Utility of Ethylene-Diamine-Tetraacetic Acid Buffer Solution With Boric Acid for Immunostaining of Specimens Stored for an Extended Period

**DOI:** 10.7759/cureus.17549

**Published:** 2021-08-29

**Authors:** Hideki Hatta, Takeshi Nishida, Takashi Minamisaka, Koichi Tsuneyama, Johji Imura

**Affiliations:** 1 Department of Diagnostic Pathology, University of Toyama, Toyama, JPN; 2 Department of Pathology and Laboratory Medicine, Tokushima University, Tokushima, JPN

**Keywords:** ethylene-diamine-tetraacetic acid, immunostaining, antigen retrieval, boric acid, old specimen, edta

## Abstract

Antigen modification and denaturation are recognized causes of false negatives in immunostaining. Specimens that have been stored for an extended period at room temperature show decreased immunoreactivity and may mislead the diagnosis. Studies of the molecular targeting of drugs often involve immunostaining of previous samples and, in some situations, only unstained specimens can be used. The present study aimed to develop an effective staining method to recover antigen activation in unstained specimens stored for an extended period by using ethylene-diamine-tetraacetic acid (EDTA) buffer solution with boric acid. We compared several commonly used antigen retrieval solutions and found that Tris-borate-EDTA (TBE) buffer solution with a pH ≥8.3 provided sufficient antigen retrieval. However, pH values higher than 8.3 (9.0, 10.0, and 11.0) frequently caused severe tissue damage. Thus, TBE with pH 8.3 was the most suitable antigen retrieval solution for recovering the antigenicity of specimens stored for an extended period. This procedure may allow useful immunohistochemical information, even from sections that have been stored for an extended period.

## Introduction

Antigen modification and denaturation are recognized causes of false-negative reactions in immunostaining. Specimens that have been stored for an extended period at room temperature show decreased immunoreactivity and may mislead the diagnosis [1-3]. When immunostaining is required as a companion test for the selection of cancer treatment, more than ten cut specimens must be initially prepared to avoid sample depletion. If sections stored for an extended period can also be used for immunostaining, the number of indications for companion testing will be expanded.

We previously reported new immunostaining techniques to enhance staining activity using microwave heating or ultrasonic emission [4-7]. However, the recovery of staining activity of old specimens was not easy, even in these advanced procedures. Recently, the effectiveness of a thermal treatment that uses boric acid buffer solution for DNA analysis with polymerase chain reaction (PCR) was reported, and the efficient separation performance of an ethylene-diamine-tetraacetic acid (EDTA) buffer solution containing Tris-salt-based borax has garnered attention as an electrophoresis buffer solution for DNA and RNA [8,9]. We hypothesized that this buffer might be effective in immunostaining. The present study aimed to develop an effective staining method that improves antigen activation of old unstained specimens by using an EDTA buffer solution with boric acid. 

## Technical report

Materials and Methods

Specimens

A formalin-fixed paraffin-embedded tissue microarray block containing various autopsied organs, including breast cancer, was prepared after approval of the ethical review (No. 3972) by the ethics committee of Tokushima University Hospital. Unstained specimens can present decreased antigenicity as early as two weeks after preparation, and the decrease becomes notable after six months [1-3]. In the present study, unstained sections obtained from a tissue microarray block were left for up to eight months in the laboratory at room temperature. The control was unstained sections that were cut from the same tissue array block and used immediately.

Antigen Retrieval Solutions

Citric acid buffer solution (pH 6.0; CB6.0) is a standard antigen retrieval solution [10]. In the current study, Tris-EDTA buffer solution (pH 9.0; TE9.0), boric acid buffer solution (pH 7.4; BB7.4), boric acid buffer solution (pH 9.0; BB9.0), and Tris-boric acid-EDTA buffer solution (pH 8.3; TBE8.3) were used.

Primary Antibodies

Mouse monoclonal antibodies against Ki-67 (neat, Nichirei Biosciences, Tokyo, Japan), p53 (neat, Nichirei Biosciences, Tokyo, Japan), ER (neat, Nichirei Biosciences, Tokyo, Japan), c-erbB2 (1:200 dilution, Nichirei Biosciences, Tokyo, Japan), and E-cadherin (neat, Nichirei Biosciences, Tokyo, Japan) were used.

Immunostaining procedure

After thermal heating using a Pascal Pressure Chamber (Agilent, Santa Clara, CA, US) in each antigen retrieval solution, a primary antibody was applied and incubated for 1 hour at room temperature [11-12]. After washing with Tris-buffered saline (TBS), a dextran polymer with secondary antibodies (EnVision+ Single Reagent (HRP. Mouse), Agilent, Santa Clara, CA, US) was applied and incubated for one hour at room temperature. 3,3'-Diaminobenzidine tetrahydrochloride (DAB) was used as the substrate for color development. 

Evaluation

Antigenicity recovery by each retrieval solution was evaluated using a scoring system. The degree of staining was scored as follows: staining of the control specimen = 3, weaker than the control but positive = 2, slightly positive = 1, and no staining = 0. Numerous pathologists objectively quantified the staining results and determined the stable combination of antigen activation methods.

Results

**Figure 1:**
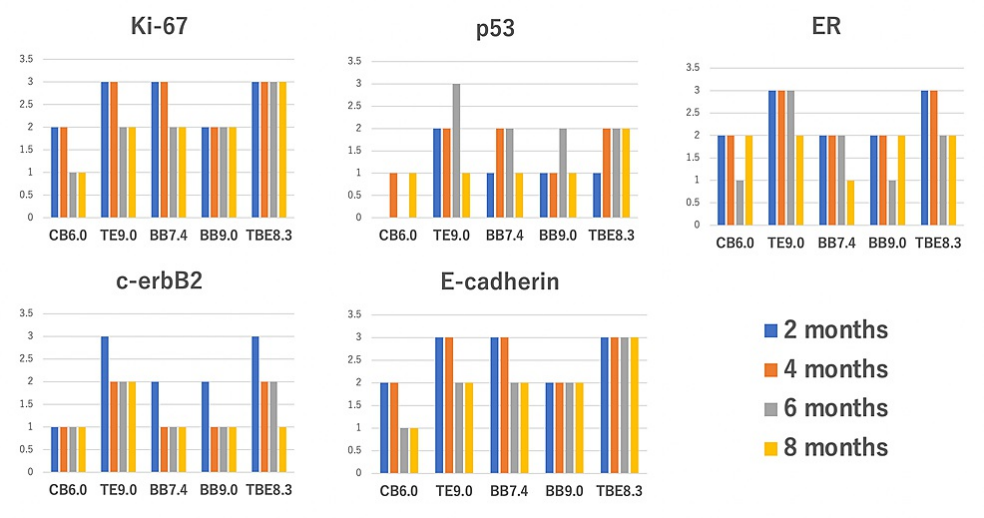
The degree of antigenicity recovery scoring Antigenicity recovery by each retrieval solution was evaluated using a scoring system. The degree of staining was scored as follows: staining of the control specimen = 3, weaker than the control but positive = 2, slightly positive = 1, and no staining = 0.

 The degree of antigenicity recovery scoring is shown in Figure 1.

**Figure 2:**
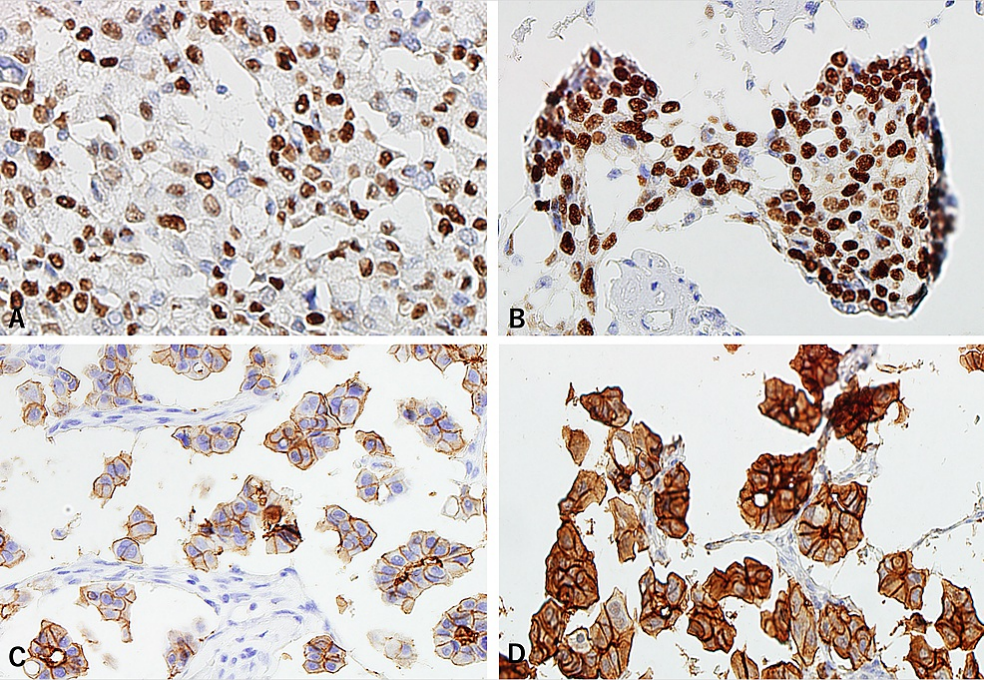
Representative staining pattern of eight-month-old specimens using solutions with pH ≥8.3. Solutions used were pH ≥8.3. (A) ER immunostaining by TBE8.3 (Score 2); (B) ER immunostaining by TBE11 (Score 3; evident severe tissue damage); (C) Immunostaining of c-erbB2 by TBE8.3 (Score 2); (D) Immunostaining of c-erbB2 by TBE11 (Score 3; evident severe tissue damage).

Briefly, the order of advantages was as follows: TBE8.3 > TE9.0 = BB7.4 > BB9.0 > CB6.0 for Ki-67, TBE8.3 ≥ TE9.0 > BB7.4 > BB9.0 > CB6.0 for p53, TE9.0 ≥ TBE8.3 > BB7.4 ≥ BB9.0 > CB6.0 for ER, TE9.0 ≥ TBE8.3 > BB7.4 = BB9.0 > CB6.0 for c-erbB, and TBE8.3 > TE9.0 = BB7.4 > BB9.0 > CB6.0 for E-cadherin. Among the buffer solutions, TBE8.3 and TE9.0 showed pronounced activation effects, and CB6.0 (which is the most commonly used buffer) displayed the lowest activation effect. We examined TBE with a pH of 9.0, 10, and 11. As expected, they showed improved activation effects, but also frequently caused severe tissue damage, such as detachment and cellular degeneration (Figure 2).

Taken together, we concluded that TBE 8.3 is the most suitable antigen retrieval solution for recovering the antigenicity of sections stored for a long period at room temperature.

## Discussion

The present study shows that the recovery of antigenicity can be expected using TBE 8.3 and TE9.0 for unstained specimens that have been left for up to eight months at room temperature. Although the mechanism of antigenicity masking in old specimens is complex and remains unclear, denaturation of the three-dimensional structure of the epitope of the target amino acid is one suggested reason [13-16]. Heating with TBE may help repair such a structural alteration. Here, TBE with pH exceeding 8.3 caused visible damage to specimens. There is currently no established antigen retrieval solution for specimens stored for a long time. The present results indicate the value of TBE 8.3 in the recovery of antigenicity of previously cut specimens. This antigen retrieval procedure may contribute to the additional immunohistochemical analysis of previous samples, which will be especially valuable when only sections are available. 

There are several reasons for false negatives in immunostaining. A more extended fixation period or higher concentration of formalin makes immunostaining difficult [17,18]. Because the preparation of paraffin blocks varies between laboratories, standardization of immunostaining quality is a concern. In further studies, we plan to examine the antigen retrieval activity of TBE 8.3 for specimens maintained for an extended period as well as for excessively fixed specimens. The degree of antigen inactivation varies depending on the method of specimen storage, and the degree of antigen retrieval is also thought to vary depending on the method of applying heating, antibody concentration, and incubation length. In our study, TBE with pH 8.3 was the optimal condition, but it is expected that different facilities will give different results. Based on the usefulness of TBE with pH 8.3, it is desired that antigen activation method suitable for each facility will be examined.

## Conclusions

Thermal heating using a pressure cooker in Tris-boric acid-EDTA buffer solution (pH 8.3) may allow useful immunohistochemical information, even from sections that have been stored for an extended period. If reliable immunostaining is available for sections stored for a long time, it is expected that more materials can be used for additional companion diagnoses.
